# Greater reliance on proprioceptive information during a reaching task with perspective manipulation among children with autism spectrum disorders

**DOI:** 10.1038/s41598-021-95349-0

**Published:** 2021-08-05

**Authors:** Masahiro Hirai, Takeshi Sakurada, Jun Izawa, Takahiro Ikeda, Yukifumi Monden, Hideo Shimoizumi, Takanori Yamagata

**Affiliations:** 1 Department of Cognitive and Psychological Sciences, Graduate School of Informatics, Nagoya University, Furo-cho, Chikusa-ku, Nagoya, 464-8601 Japan; 2 Center for Development of Advanced Medical Technology, Jichi Medical University, 3311-1 Yakushiji, Shimotsuke, Tochigi 329-0392 Japan; 3 Department of Pediatrics, Jichi Medical University, 3311-1 Yakushiji, Shimotsuke, Tochigi 329-0392 Japan; 4 Department of Robotics, College of Science and Engineering, Ritsumeikan University, 1-1-1, Noji-Higashi, Kusatsu, Shiga 525-8577 Japan; 5 Department of Neurosurgery, Jichi Medical University, 3311-1 Yakushiji, Shimotsuke, Tochigi 329-0392 Japan; 6 Faculty of Engineering, Information and Systems, University of Tsukuba, 1-1-1 Tennodai, Tsukuba, Ibaraki 305-8573 Japan; 7 International University of Health and Welfare Hospital, 537-3 Iguchi, Nasushiobara, Tochigi 329-2763 Japan; 8 University of Health and Welfare Rehabilitation Center, Nasu Institute for Developmental Disabilities, 2600-7 Kitakanamaru, Otawara, Tochigi 324-0011 Japan

**Keywords:** Human behaviour, Neurodevelopmental disorders

## Abstract

Difficulties with visual perspective-taking among individuals with autism spectrum disorders remain poorly understood. Many studies have presumed that first-person visual input can be mentally transformed to a third-person perspective during visual perspective-taking tasks; however, existing research has not fully revealed the computational strategy used by those with autism spectrum disorders for taking another person’s perspective. In this study, we designed a novel approach to test a strategy using the opposite-directional effect among children with autism spectrum disorders. This effect refers to how a third-person perspective as a visual input alters a cognitive process. We directly manipulated participants’ visual perspective by placing a camera at different positions; participants could watch themselves from a third-person perspective during a reaching task with no endpoint feedback. During a baseline task, endpoint bias (with endpoint feedback but no visual transformation) did not differ significantly between groups. However, the endpoint was affected by extrinsic coordinate information in the control group relative to the autism spectrum disorders group when the visual perspective was transformed. These results indicate an increased reliance on proprioception during the reaching task with perspective manipulation in the autism spectrum disorders group.

## Introduction

Understanding the perspective of others is a key ability when navigating the social world^1^. Specifically, it is necessary to grasp that different individuals will not view the same scene or environment in exactly the same way. Piaget and Inhelder defined this skill as ‘visual perspective-taking’ (VPT)^2^ and examined it by means of a ‘three-mountain task’. In this task, a child is shown an array of mountains and asked to describe how they would appear to a doll placed in a different location.

Subsequently, Flavell, Everett, Croft, and Flavell^3^ theorized that two levels of VPT could be delineated: Level 1 VPT (VPT1) presumes knowledge regarding the objects in one’s view that are visible to another observer, while Level 2 VPT (VPT2) presumes that two different observers can have unique visual experiences of the same scene or object. Other groups expanded this concept by showing that VPT1 and VPT2 are not acquired simultaneously. Specifically, infants first understand VPT1 from around 14 months^4, 5^ to 24 months^6^, while VPT2 comprehension can be present at 3 years of age^7^ but continues to develop over the first 11 years of life^8^. Crucially, typically developing (TD) children frequently respond to their own perspective even when required to take another person’s perspective^2, 8–14^.

Several previous studies have shown that children with autism spectrum disorders (ASD) have difficulty with VPT, particularly with tasks that involve shifting their perspective towards others’ perspectives (VPT2)^15–18^. One reason for atypical performance on the VPT task in children with ASD may be the atypical computation of theory of mind task. For example, Hamilton and colleagues directly tested the relationship between the performances on VPT2 and those on a series of false belief tasks and found a significant correlation between the two tasks^15^. Another possible reason for this is that the ability required for the VPT2 task is closely related to the ability to simulate the manipulation of one’s own bodily location in a given space^8^. Therefore, the atypical bodily processes among children with ASD may lead to atypical performance on VPT-related tasks^19^.

Another possible explanation for the difficulty children with ASD experience in grasping the concept of VPT may be that their utilization of coordinate systems to compute another person’s perspective is different from that of TD children. When encoding the external world, a representation can be perceived through either of two reference frames: egocentric or allocentric. An egocentric representation encodes the environment in reference to one’s own body, whereas an allocentric representation encodes information about landmarks in the external environment. Atypical utilization of the coordinate system among individuals with ASD has been observed in several studies. In particular, individuals with ASD tend to rely on egocentric representations to learn reaching movements towards a target rather than on allocentric representations^20, 21^.

In order to further pursue these possible explanations for the atypical relationship many ASD children have with VPT, refinements of the VPT tasks may be needed. In most previous studies with VPT tasks, participants have been instructed to answer questions based on a model’s perspective, with the model directly facing object(s) on a screen^2, 15^. This experimental arrangement presumes that first-person visual input can be transformed into a third-person perspective. Although several behavioural studies on perspective-taking tasks among children with ASD have explored differential strategies for computing the perspective of others by comparing results for other cognitive tasks between children with ASD and TD children^22, 23^, the details of these strategies remain unclear. Therefore, to resolve these issues, in this study, we introduced a novel reaching task in which participants reached for a target from a transformed visual perspective. By providing an explicit third-person perspective, we sought to learn (1) whether participants would utilize an egocentric or an allocentric coordinate system during the task and (2) how participants coordinate motor planning.

Prior to the study, we formulated two hypotheses. The first hypothesis is related to the processing of the transformed visual perspective. We hypothesized that if participants strongly relied on an egocentric coordinate system for the reaching task, and therefore had difficulty in taking the transformed-perspective visual information into account, then their reaching accuracy would not be affected even when the visual input was transformed. In contrast, if participants primarily relied on visual information, then their reaching accuracy would diminish as the transformed visual perspective information was utilized. In previous motor learning studies on individuals with ASD, participants showed a greater reliance on egocentric rather than allocentric representations^20, 21^. Therefore, we predicted that performance on the reaching task would not be affected by the transformed visual perspective among children with ASD.

The second hypothesis is related to motor coordination. We predicted that the endpoint biases might be differently affected by the target location across groups, because the difference in target location can influence the motor planning process in the joint space. In addition, in a joint space, motor planning requires the coordination of both the shoulder and elbow and is more difficult than movement the elbow alone^24^. For example, if a participant is presented a task that must be performed with the right hand, and the target is located near the participant’s right hand, then only motor planning of the elbow is required. However, when the target is located to the participants’ left side or front-centre, coordination of both shoulder and elbow would be required, and the task would be more difficult. Therefore, we presumed that the target’s location would affect the perspective-transformed visual information. Finally, because atypical motor coordination has been reported in those with ASD^25^, we expected that reaching towards a target, which requires motor planning involving the coordination of both the shoulder and elbow, would differently modulate endpoint biases across TD and ASD groups.

## Results

### Group analyses

We first analysed group differences in endpoint biases and RTs during both the baseline and perspective-transformed tasks.

#### Baseline task

##### Endpoint biases

**Figure 1:**
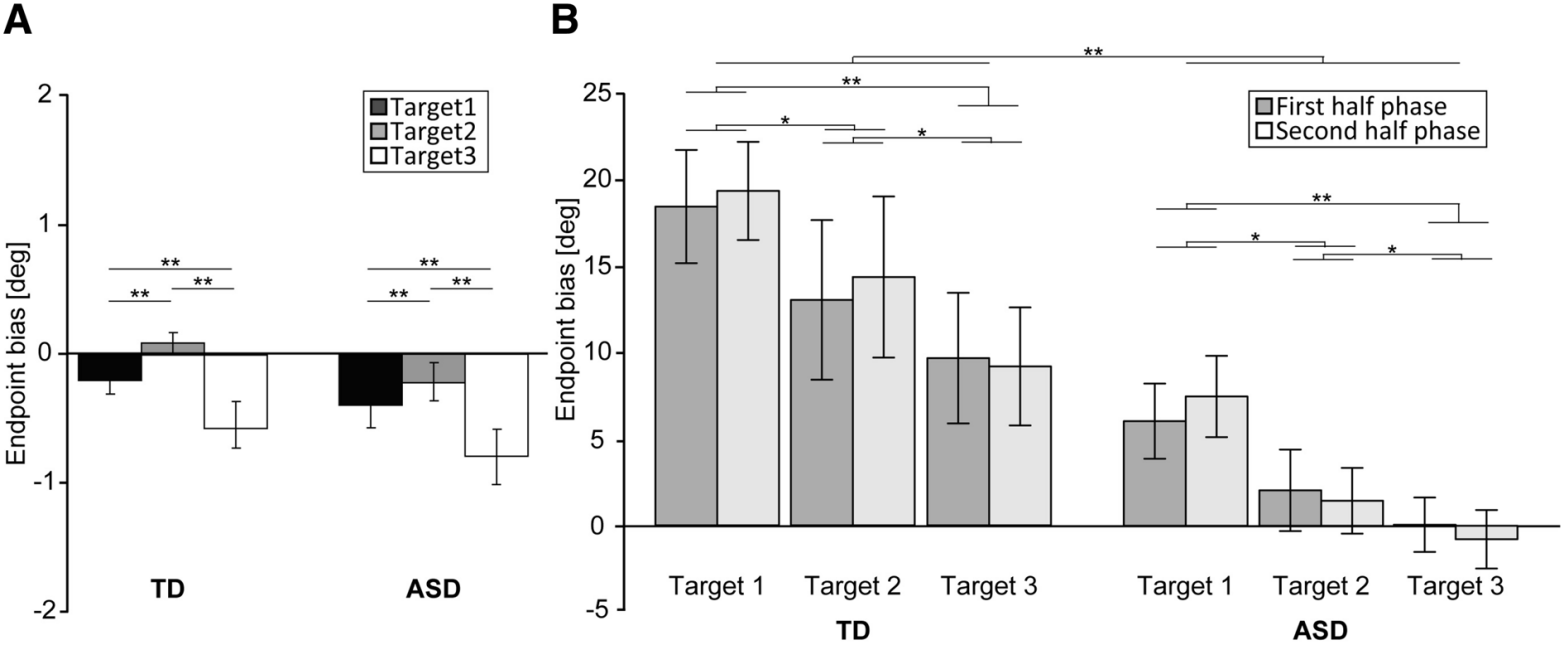
The endpoint bias (degree) in both the TD and ASD groups for both the Baseline task (**A**) and Perspective-transformed task (**B**). Error bars indicate standard errors of the mean. Typically developing group (TD); autism spectrum disorders group (ASD). *p <  0.05; **p <  0.01.

For the endpoint biases (Fig. 1A; Table 1), we found significant main effects for Target [*F*(1.82, 83.5) = 18.6, *p* < 0.001, η_*p*_^2^ = 0.11]. However, the main effect of Group [*F*(1, 46) = 1.87, *p* = 0.18, η_*p*_^2^ = 0.02] and the interaction of Group × Target [*F*(1.82, 83.5) = 0.19, *p* = 0.81, η_*p*_^2^ = 0.001] were not significant. This suggests that the endpoint biases for Target 2 were significantly larger than those for Target 1 [*t*(46) = 2.78, *p* = 0.008] and Target 3 [*t*(46) = 5.51, *p* < 0.01]. Moreover, the endpoint biases for Target 1 were significantly larger than those for Target 3 [*t*(46) = 3.56, *p* = 0.002].

**Table 1:** Endpoint bias (degree) in all phases and target locations.

Scores	Group	Target		
		Target 1 Mean (SD)	Target 2 Mean (SD)	Target 3 Mean (SD)
Baseline	ASD (*n* = 24)	− 0.40 (0.86)	− 0.22 (0.70)	− 0.80 (1.01)
	TD (*n* = 24)	− 0.21 (0.50)	0.09 (0.42)	− 0.58 (0.75)
First phase	ASD (*n* = 24)	6.10 (10.7)	2.07 (11.6)	0.06 (7.76)
	TD (*n* = 24)	18.6 (16.2)	13.1 (22.8)	9.74 (18.6)
Second phase	ASD (*n* = 24)	7.53 (11.6)	1.46 (9.38)	− 0.78 (8.34)
	TD (*n* = 24)	19.5 (14.0)	14.7 (23.0)	9.26 (16.8)

ASD: autism spectrum disorder; TD: typically developing; SD: standard deviation.

##### RTs

**Figure 2:**
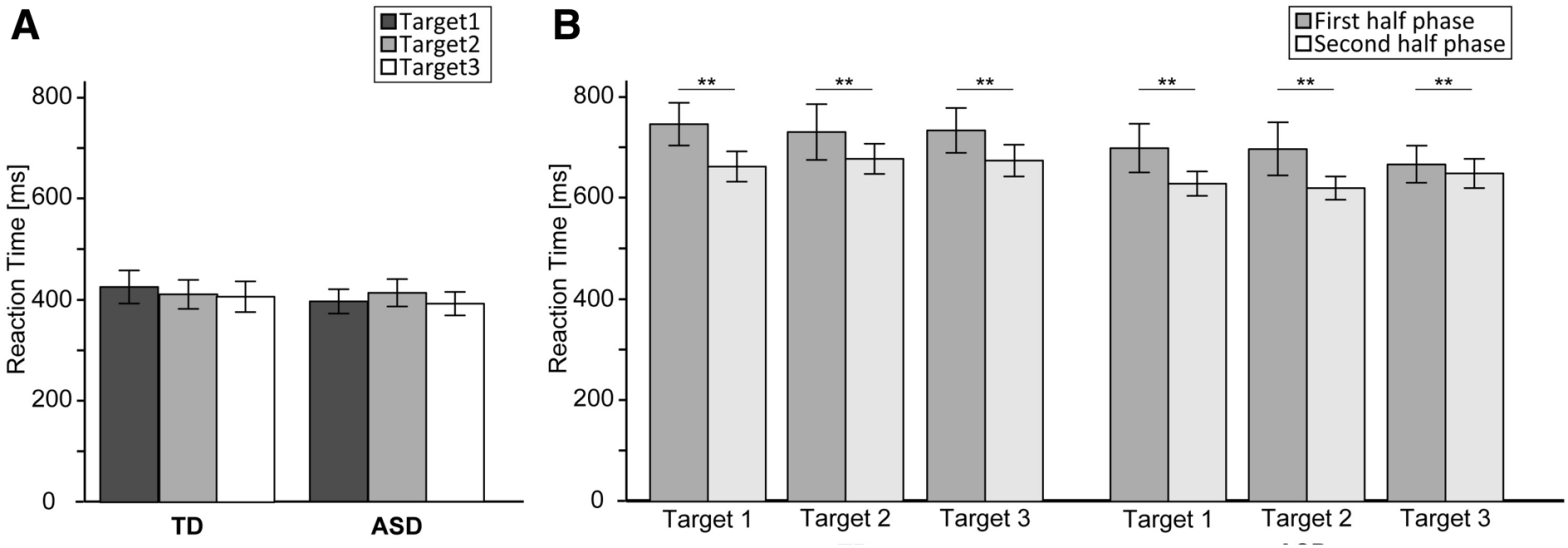
The RT (ms) in both the TD and ASD groups for both the Baseline task (**A**) and Perspective-transformed task (**B**). Error bars indicate standard errors of the mean. Reaction time (RT); Typically developing group (TD); Autism Spectrum Disorder group (ASD). **p < 0.01.

Regarding the RTs (Fig. 2A; Table 2), we did not observe significant main effects for Group [*F*(1, 46) = 0.14, *p* = 0.71, η_*p*_^2^ = 0.0031] and Target [*F*(1.65, 76.05) = 2.29, *p* = 0.18, η_*p*_^2^ = 0.005]. The two-way interaction of Group × Target was not significant [*F*(1.65, 76.05) = 2.73, *p* = 0.08, η_*p*_^2^ = 0.06].

**Table 2:** RTs (ms) in all phases and target locations.

Scores	Group	Target		
		Target 1 Mean (SD)	Target 2 Mean (SD)	Target 3 Mean (SD)
Baseline	ASD (*n* = 24)	395.2 (115.0)	412.1 (129.3)	390.6 (110.9)
	TD (*n* = 24)	425.1 (156.2)	410.5 (136.8)	405.9 (145.5)
First phase	ASD (*n* = 24)	697.8 (230.6)	695.8 (248.7)	665.4 (172.8)
	TD (*n* = 24)	745.1 (202.1)	729.5 (264.0)	732.7 (212.4)
Second phase	ASD (*n* = 24)	627.6 (115.6)	618.9 (110.3)	647.8 (138.4)
	TD (*n* = 24)	661.4 (143.2)	676.6 (143.2)	673.1 (150.2)

RTs: reaction times (ms); ASD: autism spectrum disorder; TD: typically developing; SD: standard deviation.

#### Perspective-transformed task

##### Endpoint biases

For the endpoint biases (Fig. 1B; Table 1), we found significant main effects for Group [*F*(1, 46) = 8.97, *p* = 0.004, η_*p*_^2^ =0.16] and Target [*F*(1.37, 63.13) = 14.99, *p* < 0.001, η_*p*_^2^ = 0.25]. However, the main effect of the Task phase [*F*(1, 46) = 0.08, *p* = 0.79, η_*p*_^2^ = 0.002] and the other interactions, such as Group × Target [*F*(1.37, 63.13) = 0.36, *p* = 0.62, η_*p*_^2^ = 0.001], Task phase × Target [*F*(1.83, 84.32) = 1.79, *p* = 0.18, η_*p*_^2^ = 0.004], Group × Task phase [*F*(1, 46) = 0.08, *p* = 0.78, η_*p*_^2^ = 0.002], and a three-way interaction of Group × Task phase × Target [*F*(1.83, 87.32) = 0.82, *p* = 0.43, η_*p*_^2^ = 0.02] were not significant. These results indicate that the endpoint bias of the TD group was significantly larger than those in the ASD group. Moreover, the endpoint biases for Target 1 were significantly larger than those for Target 2 [*t*(46) = 2.58, *p* = 0.02] and Target 3 [*t*(46) = 6.49, *p* < 0.001]. Further, the endpoint biases for Target 2 were significantly larger than those for Target 3 [*t*(46) = 2.64, *p* = 0.01].

##### RTs

Regarding RTs (Fig. 2B; Table 2), we observed a significant main effect of Task phase [*F*(1, 46) = 9.36, *p* < 0.01, η_*p*_^2^ = 0.17]. However, other main effects and interactions, such as Group [*F*(1, 46) = 0.85, *p* = 0.36, η_*p*_^2^ = 0.02], Target [*F*(1.81, 83.45) = 0.07, *p* = 0.91, η_*p*_^2^ = 0.002], Group × Target [*F*(1.81, 83.45) = 0.06, *p* = 0.93, η_*p*_^2^ = 0.001], Group × Task phase [*F*(1, 46) = 0.07, *p* = 0.79, η_*p*_^2^ = 0.001], Task phase × Target [*F*(1.91, 88) = 1.54, *p* = 0.22, η_*p*_^2^ = 0.03], and Group × Task phase × Target [*F*(1.91, 88) = 1.1, *p* = 0.34, η_*p*_^2^ = 0.02] were not significant. These findings suggest that the RTs in the first-half phase were significantly longer than those in the second half phase.

### Developmental trajectory analyses

As we found a significant group effect in the endpoint biases, but not RTs, we conducted a developmental trajectory analysis for the endpoint biases.

#### Endpoint biases

**Figure 3:**
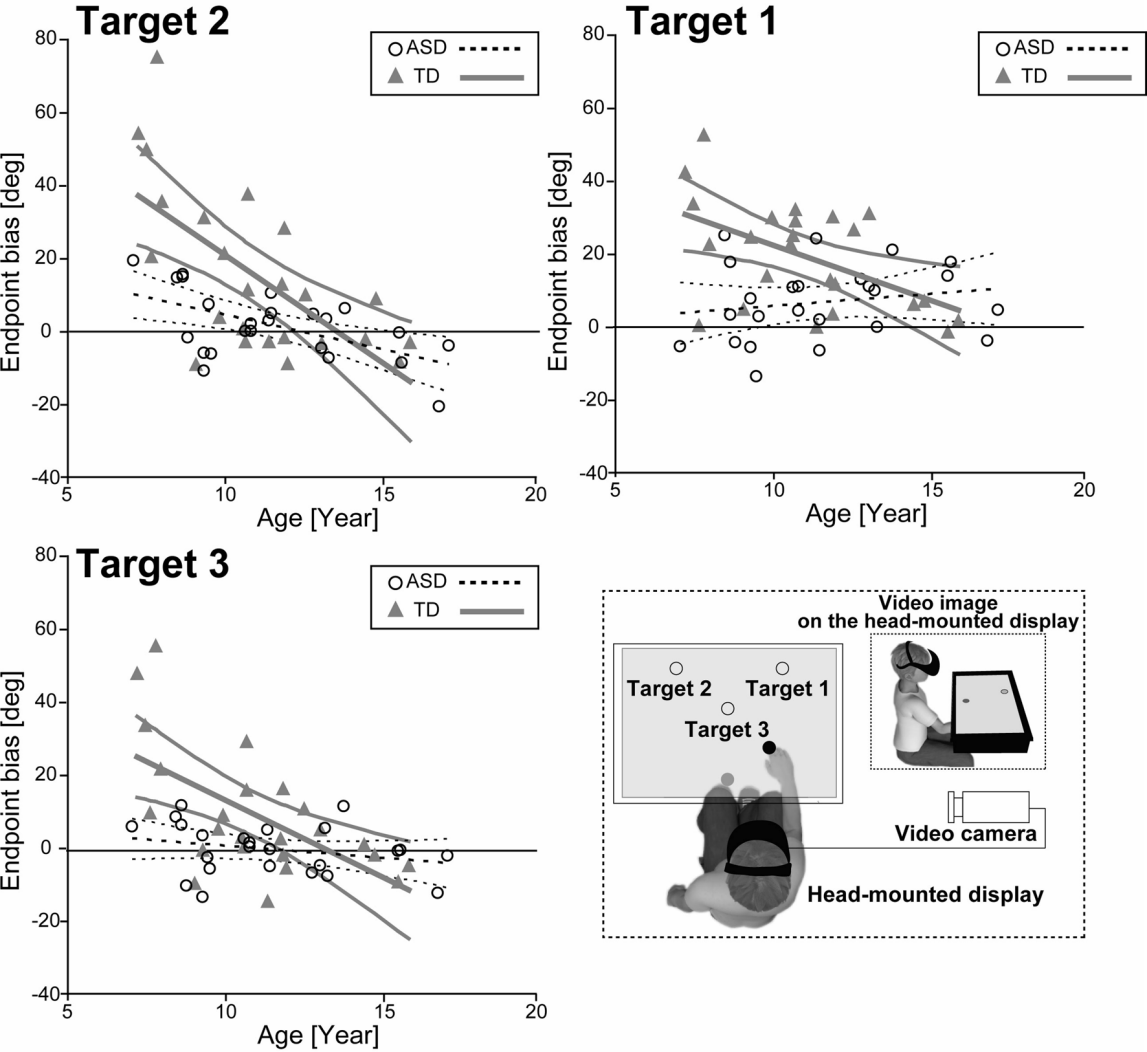
Developmental trajectories for endpoint biases during the perspective-transformed task for each target in both groups. Open circles indicate children with autism spectrum disorders (ASD) and filled triangles indicate typically developing children (TD). Dashed lines indicate the trajectories with a 95% confidence interval for the ASD group. The thick line indicates the trajectories with a 95% confidence interval for the TD group.

Given that we observed group differences in the endpoint biases during the perspective-transformed task, but not the baseline task, we further explored developmental trajectories during the perspective-transformed task (Fig. 3). Although we found speed–accuracy trade-off for the reaching performance, we did not observe any significant reduction across the phases regarding the endpoint biases, which is the index of motor learning; this was consistent with previous studies^20, 21^. Therefore, we did not divide the task into two phases as in the group analysis above.

In the developmental trajectory analysis, we added age as a covariate and conducted a developmental trajectory analysis. We found significant main effects for Age [*F*(1, 44) = 17.11, *p* < 0.001, η_*p*_^2^ = 0.280], Group [*F*(1, 44) = 14.20, *p* < 0.001, η_*p*_^2^ = 0.244], and Target [*F*(2, 88) = 9.79, *p* < 0.001, η_*p*_^2^ = 0.182]. Moreover, two-way interactions of Target × Age [*F*(2, 88) = 13.08, *p* < 0.001, η_*p*_^2^ = 0.229] and Group × Age [*F*(1, 44) = 9.55, *p* = 0.003, η_*p*_^2^ = 0.178] were significant. However, the other interactions, such as Group × Target [*F*(1.44, 63.41) = 0.192, *p* = 0.752, η_*p*_^2^ = 0.004] and a three-way interaction of Group × Age × Target [*F*(2, 88) = 0.067, *p* = 0.935, η_*p*_^2^ = 0.002] were not significant. The simple main effect of the Target × Age interaction revealed that the age effect was prominent for Target 2 [*F*(1, 46) = 21.51, *p* < 0.001, η_*p*_^2^ = 0.32] and Target 3 [*F*(1, 46) = 12.05, *p* = 0.001, η_*p*_^2^ = 0.21], but not Target 1 [*F*(1, 46) = 2.605, *p* = 0.113, η_*p*_^2^ = 0.54]. The results suggest that the endpoint bias was reduced according to age for Targets 2 and 3, but not for Target 1. Additionally, the significant interaction of Age × Group revealed that the endpoint biases of the TD group were significantly higher than in the ASD group before the age of 11.6 years.

### Correlations between questionnaire and behavioural measures

Next, we examined correlations between the bias patterns and Autism Quotient (AQ), Social Communication Questionnaire (SCQ), Japanese Picture Vocabulary test revised (PVT-R) scores, and Raven’s Coloured Progressive Matrices (RCPM) test scores (Table 3). We did not find any significant correlations after false discovery rate correction.

**Table 3:** Correlations between endpoint bias and questionnaire scores.

Scores	Group	Perspective-transformed task		
		Target 1 *r, p*	Target 2 *r, p*	Target 3 *r, p*
AQ	ASD (*n* = 24)	0.17, 0.43	0.27, 0.20	0.17, 0.43
	TD (*n* = 24)	− 0.13, 0.56	− 0.19, 0.37	− 0.23, 0.29
Current SCQ	ASD (*n* = 24)	0.07, 0.75	− 0.04, 0.85	0.06, 0.78
	TD (*n* = 24)	− 0.36, 0.08	− 0.17, 0.43	− 0.34, 0.10
Lifetime SCQ	ASD (*n* = 24)	0.04, 0.87	− 0.10, 0.63	− 0.16, 0.47
	TD (*n* = 24)	− 0.25, 0.25	− 0.10, 0.62	− 0.25, 0.24
RCPM	ASD (*n* = 24)	0.36, 0.08	− 0.43, 0.04	− 0.12, 0.58
	TD (*n* = 24)	− 0.22, 0.31	− 0.52, 0.009	− 0.39, 0.06
PVT	ASD (*n* = 24)	0.32, 0.12	− 0.54, 0.006	− 0.27, 0.21
	TD (*n* = 24)	− 0.27, 0.20	− 0.58, 0.003	− 0.54, 0.007

No significant correlations were found after false discovery rate correction.

*r*: correlation coefficient; ASD: autism spectrum disorder; TD: typically developing; SCQ: Social Communication Questionnaire; RCPM: Raven’s Coloured Progressive Matrices test; AQ: Autism Quotient; PVT: Japanese Picture Vocabulary test.

## Discussion

The results of the present experiments showed that endpoint biases in the ASD group were significantly smaller than those in the TD group when participants observed themselves from a third-person perspective. This finding suggests that, compared to the TD group, participants in the ASD group showed a greater reliance on proprioception during the reaching task with perspective transformation. In both groups, the endpoint biases decreased as age increased. Finally, although we observed both a location effect and a group effect of the endpoint biases, the endpoint biases were not differently modulated by combinations of two factors, such as target location and group. This indicates that there was no atypical motor coordination of shoulder and elbow movement in the ASD group.

We found that endpoint biases among children with ASD were less susceptible to a third-person visual perspective than those in the control group. Therefore, it seems that individuals with ASD rely relatively more on proprioceptive information than third-person visual perspective information during the reaching task. This suggests that children with ASD seem to have difficulties in putting themselves into the transformed-perspective location (i.e., imagining that their bodily location conforms with the transformed-visual perspective information provided). Given that we always provided positive feedback in the perspective-transformed task, regardless of the endpoint location, it is possible that participants did not modify their behaviour based on this feedback. This possibility was supported by the lack of any significant differences in endpoint biases in either the TD or ASD groups across the first half and second half phases during the perspective-transformed task. Therefore, the current results appear consistent with past studies regarding motor learning in children with ASD, which demonstrated that children with ASD show reliance on proprioceptive information^20, 21^.

For the ASD group, we found a greater reliance on the egocentric coordinate system than for the TD group. The reliance on proprioceptive information in the ASD group appears consistent with previous research^26–28^. For example, a series of studies on the ‘rubber hand illusion’—in which subjects watching a rubber hand being stroked while their own hand is hidden from view experience a shift in the sensed position of their own hand, mistakenly assigning it to the position of the rubber hand^29^—indicated that children with ASD are less susceptible to the illusion compared to children without ASD. In a similar vein, children with ASD performed significantly better than TD children on a heartbeat-counting task that required attention towards an inner physiological process^28^. For the TD group, this developmental profile, including a shift from a reliance on visual information to a reliance on proprioceptive information, seems to be concordant with results from studies on the rubber hand illusion^30^ and visually induced mirror illusion^31^.

In an approach similar to ours, several studies applied a prism adaptation paradigm for individuals with ASD^32, 33^, in which participants were asked to perform a motor task, such as reaching, while wearing goggles with a prism that displaced the visual field. In the prism adaptation paradigm, the visual input was a first-person perspective, which did not include the participants themselves. Contrary to our present findings, other studies reported no differences in the ability to form internal models by adopting a prism adaptation paradigm between groups of children with and without ASD^32, 33^. The discrepancy could be due to differences in the experimental paradigms between the prism adaptation studies and our current experimental paradigm. Whereas in the prism adaptation paradigm the visual image is displaced by goggles from the first-person perspective, our current experimental paradigm shifted the ‘perspective’ away from the observer. In other words, the visual scene was transformed from a first-person perspective to a third-person perspective, whereby participants were looking down at themselves and the experimental workspace from the right side of the position. Moreover, we did not provide visual feedback regarding the endpoint of the participants’ hand positions. This enabled us to explore the feedforward component of the reaching movement, rather than the ability of a child to form an internal model, which is sharpened by a feedback signal. Therefore, we accessed a different component of the reaching movement compared to previous studies that used a transformed third-person’s visual perspective.

The developmental trajectories of endpoint biases might reflect the developmental mechanisms that detect intersensory conflict between perspective-transformed visual information and proprioceptive information. Several neuroimaging studies have demonstrated that monitoring inconsistencies between visual and proprioceptive information bilaterally activate the premotor area along with the right temporoparietal junction (TPJ)^34^. Further, studies have also shown that the right posterior part of the superior temporal sulcus region^35^ and the right TPJ are critical for detecting intersensory conflict^36^. Therefore, the functional development of the right TPJ region might influence developmental changes in endpoint bias. Further, children with ASD showed less endpoint bias compared to the TD group, suggesting greater reliance on proprioceptive information in the ASD group. Thus, there is a potential relationship between developmental changes in endpoint bias and the ability to detect intersensory conflict, which may lead to smaller endpoint biases in the ASD group. However, to the best of our knowledge, no systematic research has traced the developmental changes of reliance of proprioceptive information or detecting/resolving intersensory conflict between TD and ASD. Further studies are needed to address this point.

In the current study, we observed intergroup differences in the endpoint biases for all target locations. However, we did not find a significant interaction of group and target locations in the endpoint biases. This suggests that the transformed visual information equally affected the endpoint biases and that it does not depend on the difficulty of the reaching movements.

One might think that the possibility of atypical computation of the theory of mind might have affected the current results. In fact, many previous studies have demonstrated that individuals with ASD have difficulties completing theory of mind tasks^37, 38^. Moreover, as both false belief tasks and VPT2 tasks are commonly processed in the left TPJ region^39^, it is possible that the atypical performance on the reaching task with transformed visual perspective in the ASD group was related to individuals’ abilities in theory of mind tasks. However, we did not directly test the relationship between theory of mind abilities and modulation of endpoint biases in the current study. Furthermore, we did not find any significant correlations between the endpoint biases and questionnaire scores which elucidate participants’ social functions. Contrastingly, several other studies have shown the relationship between performances on the perspective-taking task and scores on theory of mind tasks^15^. Furthermore, another study demonstrated that the reliance on proprioception is positively correlated with the severity of the child’s impairments regarding social function and imitation^20^. The discrepancies found in our study should be verified by directly testing the relationship between performances on the perspective-transformed reaching task and theory of mind tasks.

Several limitations should be acknowledged. First, our current paradigm might not have been sufficient to remove the motor learning component. We defined motor learning, based on previous studies, as the reduction of endpoint biases. Although we could not find any significant improvement in the endpoint bias across phases, we found a significant reduction in reaction time across the first and second half blocks. Therefore, although the experimental manipulation was designed to eliminate the motor learning component, it may not have sufficiently removed it. Moreover, we have provided both the endpoint feedback and reward based on participants’ performance in the baseline task to enable them to get completely used to and engage in the experimental paradigm, based on our preliminary observation. However, no information was provided in the perspective-transformed task to remove the motor learning component. The difference in the experimental procedures across tasks made it difficult to compare them. Further studies are needed to develop a paradigm in which motor planning can be directly manipulated and to remove the motor learning component completely. Second, although we found group differences in the endpoint bias when the visual perspective was rotated by 90 degrees, it remains unclear whether the modulation of the endpoint bias is linear. Our current findings could be verified by manipulating the degree to which visual information is transformed to influence the endpoint bias and RT, for example, by increasing or decreasing the angle of visual perspective. Third, although we found group differences concerning endpoint bias, the neural mechanisms remain unclear. Further neuroimaging studies are needed to identify the neural mechanisms underlying the current phenomenon. Finally, a few participants were left-handed in both groups (TD: *n* = 2, ASD: *n* = 1). Although we did not find any significant difference in their performance in the baseline task, the participants’ dexterity might have influenced the outcome regarding target accuracy. We did not measure the participants’ motor abilities in the current study. Therefore, further studies should consider how motor dexterity may affect performance.

## Conclusion

Unlike previous studies on VPT tasks, our present study directly tested how visual input from a third-person perspective can affect the performance in a reaching task without visual feedback. We directly manipulated participants’ visual perspective by placing a camera at different positions so that participants could watch themselves during the reaching task. The results revealed that children with ASD had fewer endpoint biases for this motor task compared to TD children when both groups observed themselves from a third-person perspective. The different developmental trajectory patterns observed between the two groups suggest that differential computational mechanisms underlie the performance of the reaching task when provided with transformed visual input. These findings indicate that children with ASD rely relatively highly on proprioceptive information or superior visuo-proprioceptive mapping for reaching when seeing the situation from an explicitly third-person perspective. Further studies are needed to explore the presence of a relationship between egocentrism observed in VPT tasks among children with ASD and variable use of coordinate systems during motor learning and planning.

## Methods

### Participants

Forty-eight children participated in the study. Twenty-four individuals with ASD (20 boys and 4 girls; age range = 7.1–17.2 years; *M*_age_ = 11.5 years, *SD*_age_ = 2.8) were recruited at Jichi Medical University and the International Welfare University. Twenty-four TD children (16 boys and 8 girls; age range = 7.3–15.9 years; *M*_age_ = 11.0 years, *SD*_age_ = 2.5) were recruited from nearby elementary schools, junior high schools, and high schools, to serve as the control group. We set the number of participants based on previous motor learning studies of people with ASD, which adopted similar experimental settings^20, 21^.

All children and their parents provided written, informed consent to participate. This study was approved by the ethics committees at both Jichi Medical University and International Welfare University and has been conducted in accordance with the provisions of the Declaration of Helsinki. In the TD group, 22 participants were right-handed, whereas, in the ASD group, 23 participants were right-handed, as assessed by the Edinburgh inventory^40^. Verbal mental age was assessed by the Picture Vocabulary Test-Revised (PVT-R)^41^, and nonverbal mental age was measured using Raven's Colored Progressive Matrices (RCPM) test^42^.

Mean age of the TD and matched ASD groups did not significantly differ [TD: *M*_age_ = 11.0 years, *SD*_age_ = 2.5; ASD: *M*_age_ = 11.5 years, *SD*_age_ = 2.8; *t*(46) = 0.70, *p* = 0.49], nor did the nonverbal intelligence [TD: *M* = 30.0, *SD* = 4.6; ASD: *M* = 31.0, *SD* = 4.3; *t*(46) = 0.84, *p* = 0.41] or the verbal mental age [TD: *M* = 10.6 years, *SD* = 2.0; ASD: *M* = 11.2 years, *SD* = 1.8; *t*(46) = 1.1, *p* = 0.27].

For the ASD group, ASD diagnosis was based on the *Diagnostic and Statistical Manual of Mental Disorders Fifth Edition* criteria and was confirmed by trained paediatric neurologists (T.I., Y.M., M.N., H.S., and H.W.). We used the AQ-Child^43^ (cut-off score: 25)^44^, SCQ^45, 46^, and Parent-interview ASD Rating Scale—Text Revision (PARS)^47^ to measure participants’ autistic traits. The AQ and SCQ were administered to both the TD and ASD groups; however, the PARS was only administered to the ASD group.

The PARS is a semi-structured interview in Japanese that assesses the severity of autistic symptoms^47^. It correlates significantly with scores on the Autism Diagnostic Interview-Revised^48, 49^. The PARS comprises 57 items describing symptoms of autism, with 34 relevant to behaviours observed during infancy, 33 in childhood, and 33 in adolescence and adulthood (some of the items overlap and apply to two or three of the developmental stages). In this study, the 33 childhood or adolescence/adulthood items were included in the evaluation.

The mean AQ scores for the ASD group were significantly higher than those for the TD group [TD: *M* = 11.2, *SD* = 6.0; ASD: *M* = 26.8, *SD* = 8.0; *t*(46) = 7.62, *p* < 0.001]. Current SCQ scores of the ASD group were significantly higher than of the TD group [TD: *M* = 3.9, *SD* = 3.6; ASD: *M* = 8.4, *SD* = 5.1; *t*(46) = 3.57, *p* < 0.001], as were the lifetime SCQ scores [TD: *M* = 3.2, *SD* = 3.7; ASD: *M* = 13.6, *SD* = 7.3; *t*(46) = 6.30, *p* < 0.001]. The mean PARS for 19 participants with ASD was 28.1 (*SD* = 8.0, range: 11–45). The other five participants with ASD could not be contacted for follow-up, and thus, we could not collect scores for those participants. The PARS cut-off value was nine. We confirmed that all participants were free from any neurological disorders or motor disabilities based on evaluations from five paediatric neurologists.

### Tasks and procedure

#### Apparatus for the visuomotor task

**Figure 4:**
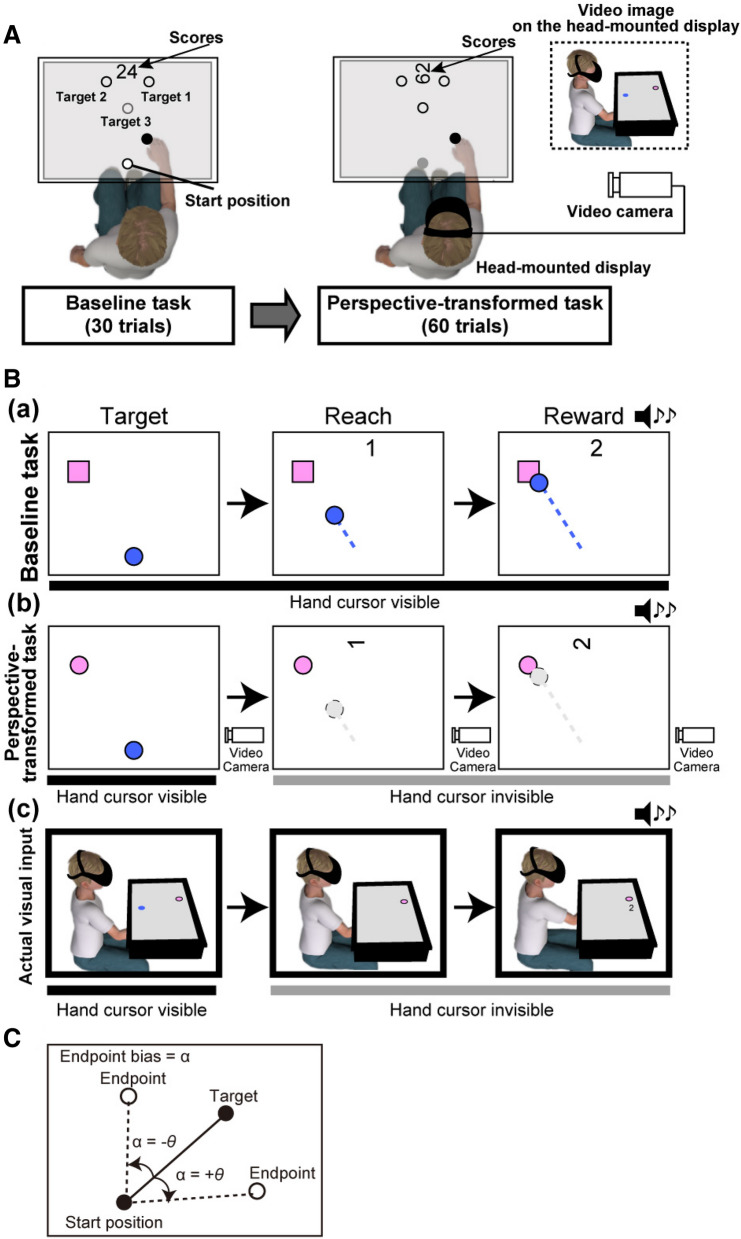
(**A**) Study setup. In both tasks, the cursor and visual target were projected on the horizontal screen, which hid the participant’s arm from their view. Baseline task: Participants were instructed to reach one of the three targets, which were displayed on the screen. The location of the right index finger was displayed on the monitor as a blue circle. Perspective-transformed task: Participants were instructed to reach one of the three targets while wearing a head-mounted display, which provided a third-person perspective recorded from the camera at the right side of the participant. The hand cursor appeared only at the ‘start’ position. Once the participant began to move his/her arm, the hand cursor disappeared. (**B**) Experimental paradigms: (a) In the baseline task, full visual feedback on the cursor position was provided, as well as the target and the sound indicating that a reach towards the target was a success or a failure; (b) In the perspective-transformed task, while the hand cursor was unseen during the hand movement, the reward signal was provided, as in the baseline task, irrespective of performance; (c) The actual visual input for participants during the perspective-transformed task; participants observed both the monitor and themselves. The numbers ‘24’ and ‘62’ in the upper part of the display represent examples of a participant’s score. (**C**) An illustration of the definition of the endpoint bias: If the endpoint is located in a counter-clockwise direction from the line between the start position and the endpoint, then the endpoint bias is negative. In contrast, if the endpoint is located in a clockwise direction from the line between the start position and the endpoint, then the endpoint bias is positive.

Each participant was seated in front of a monitor (27MP37VQ-B, LG Electronics Inc., Seoul, South Korea) placed horizontally (Fig. 4). A reflective sphere marker was attached to each participant’s right index finger (Fig. 4A). Participants’ reaching movements were performed under the monitor so that participants could not observe their hand position during the test. The movements of the right hand were tracked by a motion capture system (OptiTrack, Corvallis, OR). We simultaneously displayed the index finger’s location on the monitor using an in-house MATLAB (MathWorks, Natick, MA) code with Cogent Toolbox software (University College London, http://www.vislab.ucl.ac.uk/cogent.php). The cursor position on the monitor (hand cursor) was recorded using the toolbox at a sampling rate of 60 Hz.

#### Experimental tasks

The experiment consisted of a baseline task (30 trials) and a perspective-transformed task (60 trials). The experiment took approximately 15 to 20 min to complete. The main aim of the current experiment was to examine whether there is a group difference between children with ASD and TD children using the perspective-transformed task, rather than comparing the participants’ performances between the baseline and perspective-transformed tasks. We included the baseline task for the following reasons: first, we expected that the participants would get used to the reaching task itself during the baseline task, and second, we wished to confirm whether both groups would have similar reaching performances under a condition in which no factors were manipulated.

#### Baseline task

The purpose of the baseline task was to enable participants to get used to the experimental paradigm and motivate them to complete the task by providing entire endpoint feedback and reward based on their performances. Therefore, participants were initially instructed to place their index finger, so the cursor would be positioned at the bottom of the monitor (see Fig. 4B for the set position). After placing their index finger within the set position circle for 1 s, a target appeared in one of the three locations on the display (Fig. 4B). The target was a cartoon character, and the participant was instructed to move the circle (radius of 100 pixels, approximately 44 mm) to the visual target position as quickly and as accurately as possible when the visual target appeared. After reaching the target, feedback was given regarding how close they came to reaching the target, based on the distance between the endpoint location and the actual location to which the participant moved the circle. If the participant correctly reached the target (more precisely, when the distance between the target and the endpoint was within 100 pixels or 44 mm), then cheerful auditory feedback was given, and two points were added to the participant’s score. If the participant was close to the target (the distance between the target and the endpoint was 200 pixels or 88 mm), then neutral auditory feedback was given, and one point was added to the participant’s score. If the participant placed the circle too far from the target (the distance between the target and the endpoint was more than 200 pixels), no feedback was given, and no points were added. Each participant performed 30 trials during the baseline task.

#### Perspective-transformed task

The experimental setting was nearly identical to the baseline task, with a few notable differences. In the perspective-transformed task, participants wore a head-mounted display, which allowed a 90° perspective from the participant’s point of view (Fig. 4B). A digital video camera was placed at a height of 170 cm beside the participant. The video image was displayed via a head-mounted display (HMZ-T3W, Sony, Tokyo, Japan). The location of the cursor (i.e. right index finger) was only displayed in the initial ‘start’ position. In the perspective-transformed task, we always provided positive feedback to participants (i.e. participants earned two points for each trial, irrespective of their hand location), and participants did not receive any visual feedback regarding the endpoint of their hand movement.

The reason for this was as follows: to involve the motor learning mechanisms for the perspective transformation process, we need to provide error information, such as trajectory error or success or failure based on behaviour, to participants. However, if we always provide positive feedback to participants, no error can be detected, which will not induce the motor learning process for perspective transformation. As the reaching movement is composed of ‘feedforward’ and ‘feedback’ components, we aimed to eliminate the ‘feedback’ component by not giving visual feedback. By doing so, we expected to eliminate the effect of the motor learning for the perspective transformation process so that we could focus on the performance of motor planning. The procedure of removing visual feedback and giving positive feedback has been commonly used in previous studies^20, 21^. This method has several advantages, such as removing bias from motor planning strategy, despite the large variance in the endpoint. There were 20 trials for each of the three targets; therefore, participants completed 60 trials each. Before the experiment, we verbally confirmed that the person in the head-mounted display was actually the participant.

### Data analyses

We analysed two indices: endpoint bias and RTs. Both the endpoint bias and RTs were calculated based on methods proposed in a previous study^50^. Movement initiation was detected when the tangential velocity exceeded 40 mm/s. Movement end was defined as when the tangential velocity fell to 40 mm/s after the tangential peak velocity. As motor planning is the process of preparing motor commands to achieve the goal, it is generated before movement onset. Therefore, RTs can reflect the time taken to complete the computation of motor planning. RT was defined as the time difference between the appearance of the visual target and movement initiation. Endpoint bias was defined as the angular difference between the direction of the visual target and the endpoint of the cursor and hand movements, which is an index of motor learning (Fig. 4C).

In the analysis, we excluded trials in which the RTs were faster than 150 ms or slower than 1500 ms, following previous studies^51, 52^. Regarding the criterion for the movement trajectory, we excluded trials in which the bell-shaped velocity profile was violated while reaching as conventional arm movement should follow the profile^53, 54^. We further excluded trials in which the peak velocity did not exceed 200 mm/s; this criterion was determined based on our preliminary observations, in which the trials that were below the peak velocity did not show the bell-shaped velocity profile. Note that data from our preliminary testing, performed to determine the exclusion criteria, were not included in the current study.

Regarding the mean number of accepted trials, the mean ± standard error of the mean data were as follows: Baseline–ASD: 27.3 ± 0.7 trials, TD: 29.1 ± 0.2 trials; First half phase–ASD: 23.4 ± 1.5 trials, TD: 26.5 ± 0.8 trials; and Second half phase–ASD: 25.1 ± 1.4 trials, TD: 28.0 ± 0.5 trials. Kolmogorov–Smirnov tests were used to examine data normality. The following conditions were significant: Baseline–TD: D(24) = 0.32, *p* = 0.01; First half phase–TD: D(24) = 0.31, *p* = 0.02; Second half phase–ASD: D(24) = 0.30, *p* = 0.03, but not Baseline–ASD: D(24) = 0.27, *p* = 0.06, First half phase–ASD: D(24) = 0.19, *p* = 0.32, Second half phase–TD: D(24) = 0.26, *p* = 0.08. Thus, we utilized a Mann–Whitney U test to test the group difference in each experimental phase. We found a significant group difference in the baseline phase (signed rank = 179, *p* = 0.02), but not in the first half phase (signed rank = 238.5, *p* = 0.31) or second half phase (signed rank = 234, *p* = 0.26).

#### Baseline task

For the group analyses, we first compared endpoint bias and RTs. For the endpoint bias, a Kolmogorov–Smirnov test was used to examine data normality; no significant differences were found in any conditions (see Supplementary Materials). Therefore, we employed a mixed-design analysis of variance (ANOVA). A two-way ANOVA was applied to the endpoint bias and RT data within the participant group (ASD vs. TD), which was the between-subjects factor, and the Targets (Target 1, Target 2, and Target 3), which were the within-subject factors.

#### Perspective-transformed task

For the group analyses, we first compared endpoint bias and RTs. As confirmation was needed regarding whether the learning effect was eliminated during the test phase in the current experimental setting, we divided the perspective-transformed task into two phases and averaged the RTs and endpoint biases in the first 30 trials (first half) and the second 30 trials (second half). Note that there were no breaks between the first half and the second half of the trials during the actual perspective-transformed task.

For the endpoint bias, Kolmogorov–Smirnov tests were conducted to examine data normality. However, no significant differences were found in any conditions (see Supplementary Materials). Regarding the RTs, the Kolmogorov–Smirnov tests yielded no significant differences in any conditions (see Supplementary Materials).

As we did not find any significance in the normality of data distribution in either the endpoint bias or RTs, we applied a mixed-design ANOVA to both the endpoint bias and RTs. A two-way ANOVA was applied to the endpoint bias and RT data within the participant group (ASD vs. TD) as the between-subjects factor, and the Task phases (first half and second half) and Targets (Target 1, Target 2, and Target 3) as the within-subject factors.

We further explored the developmental trajectory approach used in previous studies^55, 56^. We constructed cross-sectional trajectories for each target’s endpoint bias during the perspective-transformed task as we found a significant group difference only in the endpoint bias. Therefore, we analysed only the endpoint biases. The Target (Target 1, Target 2, and Target 3) was used as a within-subject factor. The two groups yielded two trajectories linking endpoint bias with age. Similar to a previous study^56^, we thus added age as a covariate and performed an analysis of covariance. We calculated the point at which the upper confidence interval of the lower trajectory and the lower confidence interval of the higher trajectory overlapped, which provided an estimate of the age at which the trajectories converged. In all analyses, if the assumption of sphericity was violated, the Greenhouse–Geisser epsilon coefficient was used to correct the degrees of freedom. Both the *F-* and *p*-values were then recalculated, and statistical significance was set at *p* < 0.05.

## Supplementary Information

Supplementary Information.

## Data Availability

The datasets generated during and/or analysed during the current study are available from the corresponding author on reasonable request.

## References

[1] Frith CD (2007). The social brain?. Philos. Trans. R. Soc. Lond. B Biol. Sci..

[2] Piaget J, Inhelder B (1956). The Child's Conception of Space.

[3] Flavell JH, Everett BA, Croft K, Flavell ER (1981). Young children's knowledge about visual perception: Further evidence for the Level 1–Level 2 distinction. Dev. Psychol..

[4] Sodian B, Thoermer C, Metz U (2007). Now I see it but you don't: 14-month-olds can represent another person's visual perspective. Dev. Sci..

[5] Song HJ, Baillargeon R (2008). Infants' reasoning about others' false perceptions. Dev. Psychol..

[6] Moll H, Tomasello M (2006). Level 1 perspective-taking at 24 months of age. Br. J. Dev. Psychol..

[7] Moll H, Meltzoff AN (2011). How does it look? Level 2 perspective-taking at 36 months of age. Child Dev..

[8] Hirai M, Muramatsu Y, Nakamura M (2020). Role of the embodied cognition process in perspective-taking ability during childhood. Child Dev..

[9] Masangkay ZS (1974). The early development of inferences about the visual percepts of others. Child Dev..

[10] Flavell JH, Flavell EF, Green FL, Wilcox SA (1980). Young children’s knowledge about visual perception: Effect of observer’s distance from target on perceptual clarity of target. Dev. Psychol..

[11] Pillow BH, Flavell JH (1986). Young children’s knowledge about visual perception: Projective size and shape. Child Dev..

[12] Salatas H, Flavell JH (1976). Perspective taking: The development of two components of knowledge. Child Dev..

[13] Flavell JH (1999). Cognitive development: Children's knowledge about the mind. Annu. Rev. Psychol..

[14] Lange-Kuttner C (2009). Viewing and attention in children. Acta Pediatr..

[15] Hamilton AF, Brindley R, Frith U (2009). Visual perspective taking impairment in children with autistic spectrum disorder. Cognition.

[16] Yirmiya N, Sigman M, Zacks D (1994). Perceptual perspective-taking and seriation abilities in high-functioning children with autism. Dev. Psychopathol..

[17] Pearson A, Ropar D, Hamilton A (2013). A review of visual perspective taking in autism spectrum disorder. Front. Hum. Neurosci..

[18] Frith U, de Vignemont F (2005). Egocentrism, allocentrism, and Asperger syndrome. Conscious. Cogn..

[19] Russo L (2018). Exploring visual perspective taking and body awareness in children with autism spectrum disorder. Cogn. Neuropsychiatry.

[20] Haswell CC, Izawa J, Dowell LR, Mostofsky SH, Shadmehr R (2009). Representation of internal models of action in the autistic brain. Nat. Neurosci..

[21] Izawa J (2012). Motor learning relies on integrated sensory inputs in ADHD, but over-selectively on proprioception in autism spectrum conditions. Autism Res..

[22] Pearson A, Marsh L, Hamilton A, Ropar D (2014). Spatial transformations of bodies and objects in adults with autism spectrum disorder. J. Autism Dev. Disord..

[23] Pearson A, Marsh L, Ropar D, Hamilton A (2015). Cognitive mechanisms underlying visual perspective taking in typical and ASC children. Autism Res..

[24] Hollerbach MJ, Flash T (1982). Dynamic interactions between limb segments during planar arm movement. Biol. Cybern..

[25] Fournier KA, Hass CJ, Naik SK, Lodha N, Cauraugh JH (2010). Motor coordination in autism spectrum disorders: A synthesis and meta-analysis. J. Autism Dev. Disord..

[26] Greenfield K, Ropar D, Smith AD, Carey M, Newport R (2015). Visuo-tactile integration in autism: Atypical temporal binding may underlie greater reliance on proprioceptive information. Mol. Autism.

[27] Cascio CJ, Foss-Feig JH, Burnette CP, Heacock JL, Cosby AA (2012). The rubber hand illusion in children with autism spectrum disorders: Delayed influence of combined tactile and visual input on proprioception. Autism.

[28] Schauder KB, Mash LE, Bryant LK, Cascio CJ (2015). Interoceptive ability and body awareness in autism spectrum disorder. J. Exp. Child Psychol..

[29] Botvinick M, Cohen J (1998). Rubber hands 'feel' touch that eyes see. Nature.

[30] Cowie D, Makin TR, Bremner AJ (2013). Children's responses to the rubber-hand illusion reveal dissociable pathways in body representation. Psychol. Sci..

[31] Bremner AJ, Hill EL, Pratt M, Rigato S, Spence C (2013). Bodily illusions in young children: Developmental change in visual and proprioceptive contributions to perceived hand position. PLoS ONE.

[32] Gidley Larson JC, Bastian AJ, Donchin O, Shadmehr R, Mostofsky SH (2008). Acquisition of internal models of motor tasks in children with autism. Brain.

[33] Mostofsky SH, Bunoski R, Morton SM, Goldberg MC, Bastian AJ (2004). Children with autism adapt normally during a catching task requiring the cerebellum. Neurocase.

[34] Balslev D, Nielsen FA, Paulson OB, Law I (2005). Right temporoparietal cortex activation during visuo-proprioceptive conflict. Cereb. Cortex.

[35] Leube DT (2003). The neural correlates of perceiving one's own movements. Neuroimage.

[36] Papeo L, Longo MR, Feurra M, Haggard P (2010). The role of the right temporoparietal junction in intersensory conflict: Detection or resolution?. Exp. Brain Res..

[37] Baron-Cohen S, Leslie AM, Frith U (1985). Does the autistic child have a "theory of mind"?. Cognition.

[38] Tager-Flusberg H (1992). Autistic children's talk about psychological states: Deficits in the early acquisition of a theory of mind. Child Dev..

[39] Schurz M, Aichhorn M, Martin A, Perner J (2013). Common brain areas engaged in false belief reasoning and visual perspective taking: A meta-analysis of functional brain imaging studies. Front. Hum. Neurosci..

[40] Oldfield RC (1971). The assessment and analysis of handedness: The Edinburgh inventory. Neuropsychologia.

[41] Ueno K, Nagoshi S, Konuki S (2008). PVT-R Kaiga goi hattatsu kensa [Picture Vocabulary Test-Revised].

[42] Sugishita M, Yamazaki K (1993). Raven's Coloured Progressive Matrices.

[43] Auyeung B, Baron-Cohen S, Wheelwright S, Allison C (2008). The autism spectrum quotient: Children's version (AQ-Child). J. Autism Dev. Disord..

[44] Wakabayashi A (2007). The Autism-Spectrum Quotient (AQ) Japanese children's version: Comparison between high-functioning children with Autism Spectrum Disorders and normal controls. Shinrigaku Kenkyu.

[45] Lord C (2000). The autism diagnostic observation schedule-generic: A standard measure of social and communication deficits associated with the spectrum of autism. J. Autism Dev. Disord..

[46] Rutter M, Bailey A, Lord C (2003). The Social Communication Questionnaire Manual.

[47] Ito H (2012). Validation of an interview-based rating scale developed in Japan for pervasive developmental disorders. Res. Autism Spectr. Disord..

[48] Le Couteur A (1989). Autism diagnostic interview: A standardized investigator-based instrument. J. Autism Dev. Disord..

[49] Lord C, Rutter M, Le Couteur A (1994). Autism Diagnostic Interview-Revised: A revised version of a diagnostic interview for caregivers of individuals with possible pervasive developmental disorders. J. Autism Dev. Disord..

[50] Saijo N, Gomi H (2012). Effect of visuomotor-map uncertainty on visuomotor adaptation. J. Neurophysiol..

[51] Ratcliff R (1993). Methods for dealing with reaction time outliers. Psychol. Bull..

[52] Hultsch DF, MacDonald SW, Dixon RA (2002). Variability in reaction time performance of younger and older adults. J. Gerontol. B Psychol. Sci. Soc. Sci..

[53] Abend W, Bizzi E, Morasso P (1982). Human arm trajectory formation. Brain.

[54] Flash T, Hogan N (1985). The coordination of arm movements: An experimentally confirmed mathematical model. J. Neurosci..

[55] Annaz D, Karmiloff-Smith A, Johnson MH, Thomas MS (2009). A cross-syndrome study of the development of holistic face recognition in children with autism, Down syndrome, and Williams syndrome. J. Exp. Child Psychol..

[56] Thomas (2009). Using developmental trajectories to understand developmental disorders. J. Speech. Lang. Hear. Res..

