# Supplementary material for: Greater reliance on proprioceptive information during a reaching task with perspective manipulation among children with autism spectrum disorders
Source: Sci Rep. 2021 Aug 5;11:15974. doi: 10.1038/s41598-021-95349-0 (PMC8342420; doi:10.1038/s41598-021-95349-0)
Supplement: Supplementary file 1 — Supplementary Information. [file 41598_2021_95349_MOESM1_ESM.docx]

**Supplementary Information**

**Greater Reliance on Proprioceptive Information During a Reaching Task with**

**Perspective Manipulation Among Children with Autism Spectrum Disorders**

Masahiro Hirai, Takeshi Sakurada, Jun Izawa, Takahiro Ikeda, Yukifumi Monden,

Hideo Shimoizumi, Takanori Yamagata

1. ***Data Analyses***
   1. *Baseline task*

For the group analyses, we first compared endpoint bias and RTs. For the endpoint bias, a Kolmogorov–Smirnov test was used to examine data normality. No significant differences were found in any conditions: Baseline–ASD [Target 1: *D*(24) = 0.13, *p = .*79; Target 2: *D*(24) = 0.15, *p = .*60; Target 3: *D*(24) = 0.13, *p = .*71]; TD [Target 1: *D*(24) = 0.10, *p = .*94; Target 2: *D*(24) = 0.11, *p = .*92; Target 3: *D*(24) = 0.15, *p = .*62]. Regarding the RTs, the results of the Kolmogorov–Smirnov test did not yield any significant differences in any conditions: Baseline-ASD [Target 1: *D*(24) = 0.09, *p = .*98; Target 2: *D*(24) = 0.16, *p = .*57; Target 3: *D*(24) = 0.11, *p = .*92]; TD [Target 1: *D*(24) = 0.13, *p = .*80; Target 2: *D*(24) = 0.11, *p = .*89; Target 3: *D*(24) = 0.11, *p = .*89].

- 1. *Perspective-transformed task*

For the endpoint bias, Kolmogorov–Smirnov tests were conducted to examine data normality. However, no significant differences were found in any conditions: First half phase–ASD [Target 1: *D*(24) = 0.11, *p = .*91; Target 2: *D*(24) = 0.14, *p = .*70; Target 3: *D*(24) = 0.10, *p = .*94], TD [Target 1: *D*(24) = 0.15, *p = .*61; Target 2: *D*(24) = 0.19, *p = .*33; Target 3: *D*(24) = 0.20, *p = .*25]; Second half phase–ASD [Target 1: *D*(24) = 0.10, *p = .*95; Target 2: *D*(24) = 0.11, *p = .*91; Target 3: *D*(24) = 0.09, *p = .*97], TD [Target 1: *D*(24) = 0.13, *p = .*79; Target 2: *D*(24) = 0.18, *p = .*36; Target 3: *D*(24) = 0.13, *p = .*80].

Regarding the RTs, the Kolmogorov–Smirnov tests yielded no significant differences in any conditions: First half phase–ASD [Target 1: *D*(24) = 0.26, *p = .*09; Target 2: *D*(24) = 0.19, *p = .*29; Target 3: *D*(24) = 0.25, *p = .*09], TD [Target 1: *D*(24) = 0.21, *p = .*22; Target 2: *D*(24) = 0.25, *p = .*08; Target 3: *D*(24) = 0.13, *p = .*82]; Second half phase–ASD [Target 1: *D*(24) = 0.13, *p = .*81; Target 2: *D*(24) = 0.16, *p = .*53; Target 3: *D*(24) = 0.18, *p = .*40], TD [Target 1: *D*(24) = 0.17, *p = .*45; Target 2: *D*(24) = 0.22, *p = .*16; Target 3: *D*(24) = 0.18, *p = .*36].

1. ***Data Analyses (including a task as a within-subject factor)***

For the group analyses, we first compared both endpoint biases and RTs using a mixed-design repeated-measures analysis of variance (ANOVA) with Greenhouse-Geisser epsilon corrections for non-sphericity. A three-way ANOVA was applied to the endpoint bias and RT data within the participant group (ASD vs. TD) as a between-subjects factor and Task (Baseline, Test [first-half phase], Test [second-half phase]) and Target (Target 1, Target 2, and Target 3) as within-subject factors.

1. ***Group Analyses***

We first analysed group differences in endpoint biases and RTs during both the baseline and perspective-transformed tasks.

- 1. *Endpoint biases*

For the endpoint biases, we found significant main effects of Group, *F*(1, 46) = 9.15, *p* = .004, η*_p_^2^* = .17, Task, *F*(1.43, 65.59) = 17.35, *p* < .001, η*_p_^2^* = .27, and Target, *F*(1.37, 63.22) = 15.51, *p* < .001, η*_p_^2^* = .25. Moreover, the two-way interactions of Task × Target, *F*(2.12, 97.79) = 11.15, *p* < .001, η*_p_^2^* = .20, and Group × Task, *F*(1.43, 65.59) = 6.98, *p* = .005, η*_p_^2^* = .13, were significant. However, the other interactions were not (*F*s < 0.45; *p*s > .64).

The significant interaction of Group × Task suggests that the endpoint bias across tasks was significant in the TD group, *F*(1.21, 27.93) = 14.79, *p* < .001, η*_p_^2^* = .39, but not the ASD group, *F*(2, 45.97) = 2.68, *p* = .08, η*_p_^2^* = .10. In the TD group, the endpoint biases in the first-[t(23) = 3.72, p = 0.011] and second-half [t(23) = 4.28, p = 0.0003] phases of perspective-transformed task were significantly higher than in the baseline task. Moreover, the endpoint biases in the TD group were significantly higher than those in the ASD group during the first-[*F*(1, 46) = 7.30, *p* < .01, η*_p_^2^* = 0.137] and second-half [*F*(1, 46) = 9.54, *p* = .003, η*_p_^2^* = 0.172] phases of the perspective-transformed task, but not in the baseline task [*F*(1, 46) = 1.87, *p* = .18, η*_p_^2^* = .039. This suggests that the endpoint in the TD group was shifted towards the location of the camera, implying that the transformed visual perspective affected motor planning in the TD group more than in the ASD group.

As for the significant interaction of Task × Target, the endpoint biases in the first- and second-half phases of the perspective taking were significantly higher than those in the baseline phase across all targets [Target 1: first-half phase *t*(46) = 6.41, *p* < .001, second-half phase *t*(46) = 7.46, *p* < .001; Target 2: first-half phase *t*(46) = 2.94, *p* < .01, second-half phase *t*(46) = 3.16, *p* < .01; Target 3: first-half phase *t*(46) = 2.70, *p* = .01, second-half phase *t*(46) = 2.58, *p* = .01]. Moreover, the endpoint biases at Target 1 [*t*(46) = 5.51, *p* < .001] and 2 [*t*(46) = 3.55, *p* < .001] were significantly higher than those at Target 3, and the endpoint bias at Target 2 was significantly higher than that at Target 1 [*t*(46) = 2.78, *p* = .01] during the baseline task. During both first- and second-half phases, the endpoint biases at Target 1 were significantly higher than those at Targets 2 [first-half phase: *t*(46) = 2.45, *p* = .02; second-half phase: *t*(46) = 2.58, *p* = .01] and 3 [first-half phase: *t*(46) = 5.33, *p* < .001; second-half phase: *t*(46) = 7.04, *p* < .001]. Moreover, the endpoint bias at Target 2 was significantly higher than that at Target 3 [first-half phase: *t*(46) = 2.70, *p* = .03; second-half phase: *t*(46) = 2.54, *p* = .01].

- 1. *RTs*

Regarding RTs, we observed a significant main effect of Task, *F*(1.6, 73.66) = 134.87, *p* < .001, η*_p_^2^* = .746. This suggests that RTs during the perspective-transformed task in the first-[*t*(46) = 13.00, *p* < 0.001] and second-half [*t*(46) = 16.52, *p* < 0.001] phases were significantly longer than during the baseline task. Moreover, the RTs during the first-half phase were significantly shorter than those during the second-half phase [*t*(46) = 3.06, *p* = 0.004]. No other main effects or interactions were significant (*F*s < 1.40, *p*s > .25).
